# Diagnostic Performance of BMI and Waist Circumference in Detecting Excess Adiposity

**DOI:** 10.1002/oby.70150

**Published:** 2026-02-22

**Authors:** John C. Lin, Clara E. Tandar, Simar S. Bajaj, Fatima C. Stanford

**Affiliations:** ^1^ Perelman School of Medicine University of Pennsylvania Philadelphia Pennsylvania USA; ^2^ Warren Alpert Medical School Brown University Providence Rhode Island USA; ^3^ Stanford University School of Medicine Stanford California USA; ^4^ Neuroendocrine Unit, Nutrition Obesity Research Center (NORCH), MGH Weight Center Massachusetts General Hospital Boston Massachusetts USA; ^5^ Departments of Medicine and Pediatrics, Harvard Medical School Harvard University Boston Massachusetts USA

## Abstract

**Objective:**

This study compared the diagnostic performance of BMI, waist circumference (WC), and Lancet Commission (LC) criteria for assessing excess adiposity measured by dual‐energy X‐ray absorptiometry (DEXA) in US adults.

**Methods:**

Using 2011–2018 NHANES data, we included 10,747 adults aged 20–59 years with relevant data. We evaluated the diagnostic performance (sensitivity, specificity, positive and negative predictive values [PPV, NPV], area under the curve [AUC]) of BMI thresholds, waist‐based measures, and LC criteria for excess adiposity, as defined by DEXA.

**Results:**

The weighted prevalence of DEXA‐defined excess adiposity was 36.4%. CDC BMI thresholds demonstrated 74.6% sensitivity and 82.2% specificity (PPV, 67.0%; NPV, 87.0%). Waist‐based measures showed higher sensitivity and lower specificity than BMI; NHLBI WC thresholds produced 88.5% sensitivity and 66.7% specificity. LC criteria performed variably: BMI plus ≥ 1 waist measure had 74.6% sensitivity and 82.2% specificity, whereas ≥ 2 waist measures had 88.5% sensitivity and 66.8% specificity. The AUC values were 0.876 for BMI, 0.882 for WC, 0.887 for waist to height ratio, and 0.715 for waist to hip ratio.

**Conclusions:**

Waist‐based definitions were more sensitive than BMI, whereas BMI provided greater specificity. LC criteria performed comparably to existing definitions. Incorporating WC into screening may improve the detection of excess adiposity.

## Study Importance

- What is already known?
  ○ BMI is widely used to define obesity. However, BMI is an inferior prognosticator of central adiposity and cardiometabolic risk compared to waist circumference (WC).
  ○ New guidelines, such as the 2025 Lancet Commission criteria, propose integrating BMI and WC for better adiposity assessment.
- What does this study add?
  ○ WC‐based definitions were more sensitive but less specific than BMI for detecting excess body fat; race‐specific BMI thresholds did not improve diagnostic accuracy.
  ○ The Lancet Commission criteria performed similarly to current BMI and WC thresholds.
- How might these results change the direction of research or focus of clinical practice?
  ○ These results support the incorporation of WC alongside BMI in obesity screening and encourage further validation of combined anthropometric criteria across diverse populations.

## 1 Introduction

Obesity affects more than 100 million US adults and is a major contributor to cardiometabolic disease and mortality [1]. Although body mass index (BMI) has historically served as the primary clinical tool for diagnosing obesity, BMI does not directly measure adiposity and does not capture variation in body fat distribution, lean mass, or population differences by age, sex, and ethnicity [2]. In recognition of these limitations, the 2025 Lancet Commission (LC) proposed redefining obesity to require evidence of excess adiposity, quantified by direct methods such as dual‐energy X‐ray absorptiometry (DEXA) or by validated surrogate markers including BMI, waist circumference (WC), and related anthropometric measures [2]. This framework shifts the focus from weight‐based thresholds to a more biologically grounded assessment of body fat, particularly as DEXA was identified as a gold standard test for obesity in American Heart Association (AHA) guidelines on assessing adiposity [3].

Despite new evidence supporting waist‐based and composite measures, they are underutilized in routine clinical practice relative to BMI [3]. Key uncertainties remain regarding the accuracy of these waist‐based and composite measures when evaluated against new composite measures of obesity proposed by the LC. A recent study reported that BMI had sensitivity of 98.4% for identifying excess adiposity, which was defined as elevated BMI combined with either elevated WC or elevated DEXA‐derived body fat percentage [4]. However, this approach misapplied the LC criteria by requiring elevated BMI to establish excess adiposity, thereby precluding an accurate assessment of BMI's diagnostic performance. Moreover, essential metrics such as specificity and positive predictive value were not reported, limiting the ability to determine whether existing BMI thresholds accurately distinguish individuals with and without excess adiposity.

To address these gaps, we used data from the National Health and Nutrition Examination Survey (NHANES) to evaluate the diagnostic performance of BMI, waist measures, and each LC composite measure for identifying DEXA‐measured excess adiposity among US adults.

## 2 Methods

This study was deemed exempt by the University of Pennsylvania Institutional Review Board guidelines. We used data from the 2011 to 2018 NHANES, a nationally representative survey of the civilian, noninstitutionalized US population conducted by the Centers for Disease Control and Prevention (CDC). Participants completed in‐home interviews and physical exams using standardized protocols. The CDC calculated BMI using weight and height. It measured WC above the iliac crest at the midaxillary line and hip circumference (HC) around the hips at the level of the maximum extension of the buttocks. We computed waist to height ratio (WHtR) and waist to hip ratio (WHR) accordingly. The NHANES response rate ranged from 49% to 70% for the included years [5].

**FIGURE 1:**
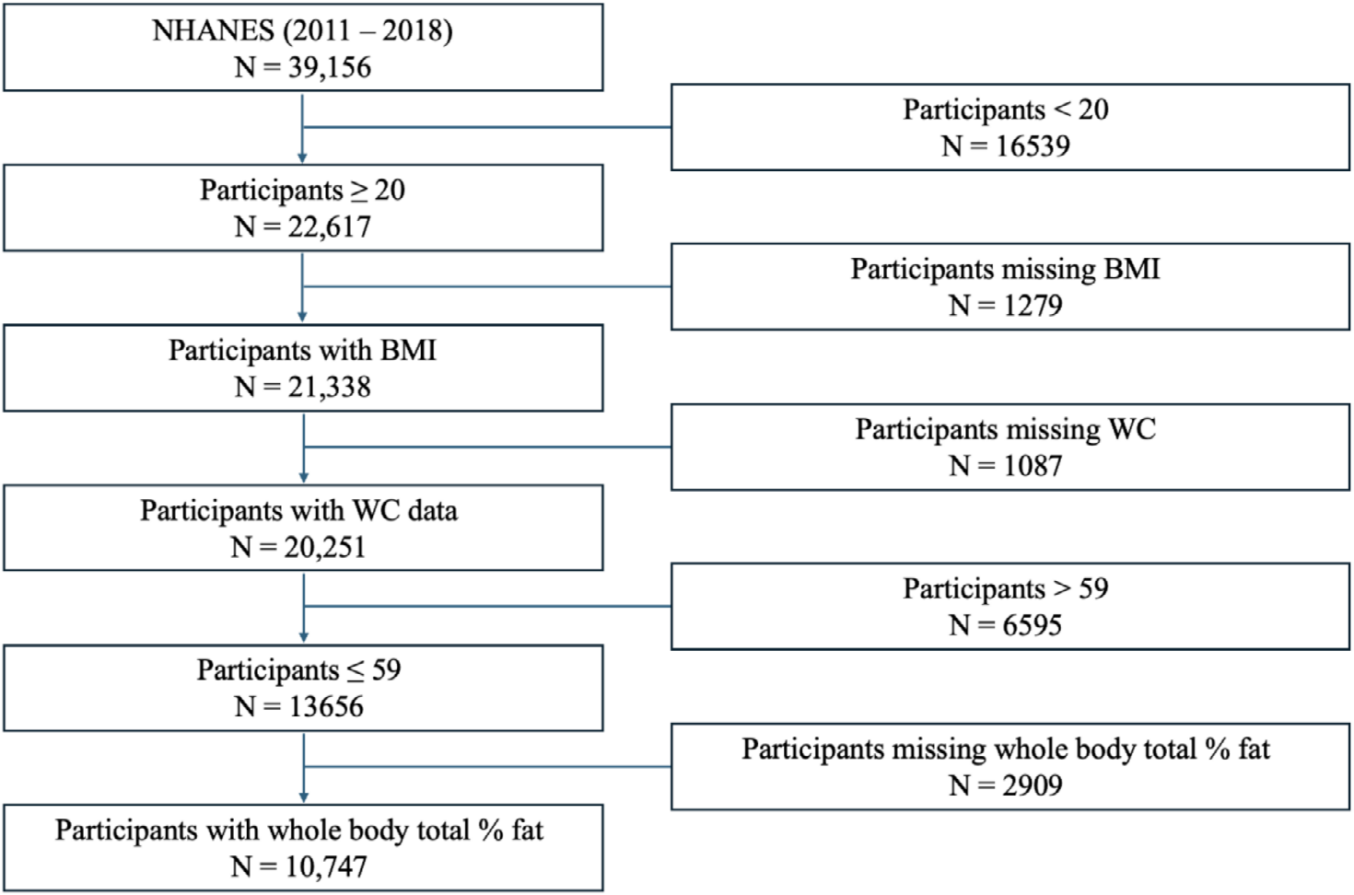
Flowchart of participants included in the study. [Color figure can be viewed at wileyonlinelibrary.com]

Our analytic sample included adults aged 20–59 years with valid data for BMI, WC, WHtR, WHR, and whole‐body DEXA scans. Individuals aged 60 years or older did not undergo a DEXA scan and were excluded. Participants with missing or invalid measurements were excluded, as shown in Figure 1, in accordance with Standards for Reporting of Diagnostic Accuracy Studies (STARD) guidelines.

Table 1 shows the definitions of obesity measures and their sources. Participants were classified as having excess adiposity if they had high body fat per DEXA (≥ 30% in men and ≥ 42% in women), as DEXA was identified as a gold standard test for obesity in AHA guidelines [2, 3]. We compared a comprehensive set of independent anthropometric measures used to define obesity. These included the CDC BMI thresholds (BMI ≥ 30) and race‐specific BMI thresholds (BMI ≥ 27.5 for Asians and ≥ 30 for all others) [6, 7]. For central adiposity, we evaluated three WC criteria: the National Heart, Lung, and Blood Institute (NHLBI) sex‐specific thresholds (WC ≥ 102 cm for men and ≥ 88 cm for women), the International Diabetes Federation (IDF) race‐ and sex‐specific thresholds (WC ≥ 90 cm for Asian men, ≥ 94 cm for other men, and ≥ 80 cm for women), the National Institute for Health and Care Excellence (NICE) WHtR thresholds (WHtR ≥ 0.50), and the Swainson et al. [11] WHtR thresholds (WHtR ≥ 0.53) [8, 9, 10, 11]. We also incorporated the World Health Organization (WHO) sex‐specific WHR thresholds (WHR ≥ 0.90 for men and ≥ 0.85 for women), which were only available for 2017–2018 [12]. Therefore, WHR thresholds were not used in composite metrics due to their limited data availability. We operationalized the LC criteria as follows: BMI plus one waist measure, defined as meeting the CDC BMI thresholds in addition to either the NHLBI WC thresholds or the NICE WHtR thresholds; and two waist measures, defined as meeting both the NHLBI WC and the NICE WHtR thresholds [2].

**TABLE 1:** Definitions and sources for different obesity measures based on national and international standards.

Obesity measures	Thresholds	Source
CDC BMI thresholds	BMI ≥ 30	[6]
Race‐based BMI thresholds	BMI ≥ 27.5 (Asians), BMI ≥ 30 (others)	[7]
NHLBI sex‐based WC thresholds	WC ≥ 102 cm (men), WC ≥ 88 cm (women)	[8]
IDF race‐ and sex‐based WC thresholds	WC ≥ 90 cm (Asian men), ≥ 94 cm (other men), ≥ 80 cm (women)	[9]
NICE WHtR thresholds	WHtR ≥ 0.50	[10]
WHtR thresholds per Swainson et al. [11]	WHtR ≥ 0.53	[11]
WHO sex‐based WHR thresholds	WHR ≥ 0.90 (men), WHR ≥ 0.85 (women)	[12]
Lancet Commission criteria		
High body fat per DEXA	≥ 30% (men), ≥ 42% (women)	[13]
BMI + ≥ 1 waist‐based measure	BMI ≥ 30 *AND* WC ≥ 102 cm (men), WC ≥ 88 cm (women) OR	[2]
≥ 2 waist‐based measures	WC ≥ 102 cm (men), WC ≥ 88 cm (women) *AND* WHtR ≥ 0.5	[2] [10]

Abbreviations: DEXA, dual‐energy X‐ray absorptiometry; WC, waist circumference; WHtR, waist to height ratio; WHR, waist to hip ratio.

We estimated the prevalence of obesity and quantified the diagnostic performance of each obesity metric by calculating sensitivity, specificity, positive predictive value (PPV), and negative predictive value (NPV) using SPSS Version 30.0.0 (IBM, Armonk, NY). Diagnostic performance was further evaluated within subgroups defined by race/ethnicity and sex. All analyses incorporated NHANES sample weights and design‐based variance estimates to account for the complex survey structure. In addition, we generated receiver operating characteristic (ROC) curves for BMI, WC, and combined anthropometric models using DEXA‐defined excess adiposity as the criterion standard.

## 3 Results

A total of 10,747 adults were included in the study. After weighting, the distribution was as follows: 34.6% were non‐Hispanic White, 25.4% Hispanic, 21.0% Black, and 14.9% Asian. Additionally, 50.1% were female.

Table 2 presents the diagnostic performance of each anthropometric measure of obesity. The weighted prevalence of DEXA‐defined excess adiposity was 36.4%. Using CDC BMI thresholds, sensitivity was 74.6% and specificity was 82.2%, with PPV of 67.0% and NPV of 87.0%. Race‐based BMI thresholds yielded similar results (sensitivity, 75.6%; specificity, 81.5%). Measures of central adiposity showed higher sensitivity and lower specificity relative to BMI‐based definitions. NHLBI WC thresholds yielded 88.5% sensitivity and 66.7% specificity, and IDF race‐based WC criteria produced 98.5% sensitivity and 39.9% specificity. WHtR‐based thresholds followed a similar pattern: NICE WHtR thresholds (≥ 0.50) had 99.5% sensitivity and specificity 33.0%; the Swainson WHtR cut point showed sensitivity 98.09% and specificity 49.19%. WHO WHR thresholds demonstrated 90.8% sensitivity and 42.1% specificity.

**TABLE 2:** Diagnostic test performance of different obesity measures for excess adiposity confirmed by DEXA.

Obesity measures^a^ ^,^ ^b^ (*N* = 10,747)	Sensitivity, %	Specificity, %	PPV, %	NPV, %
CDC BMI thresholds^c^	74.62	82.18	67.02	86.96
Race‐based BMI thresholds^d^	75.63	81.51	66.51	87.32
NHLBI WC thresholds^e^	88.54	66.74	56.38	92.31
IDF race‐based WC thresholds^f^	98.47	39.93	44.31	98.17
Swainson WHtR data (WHtR ≥ 0.53)^g^	99.52	32.96	41.88	99.30
WHO WHR thresholds^h^	90.78	42.10	44.39	89.96
Lancet Commission criteria^i^ ^,^ ^j^				
≥ 1 anthropometric measure + BMI^g^	74.62	82.18	67.02	86.96
≥ 2 anthropometric measures^h^	88.54	66.78	56.40	92.31

Abbreviations: DEXA, dual‐energy X‐ray absorptiometry; NPV, negative predictive value; PPV, positive predictive value; WC, waist circumference; WHR, waist to hip ratio; WHtR, waist to height ratio.

^a^ Excess adiposity was defined as a high percentage of body fat on DEXA: ≥ 30% (men), ≥ 42% (women).

^b^ All analyses were conducted using NHANES sample weights and variance estimates.

^c^ We used the CDC definition of obesity, BMI ≥ 30.

^d^ We used race‐based modifications per WHO recommendations; BMI ≥ 27.5 (Asians), BMI ≥ 30 (others).

^e^ We used the NHLBI WC cutoffs: WC ≥ 102 cm (men), WC ≥ 88 cm (women).

^f^ We used the IDF WC cutoffs: WC ≥ 90 cm (Asian men), ≥ 94 cm (other men), ≥ 80 cm (women).

^g^ We used the NICE WHtR cutoffs: WHtR ≥ 0.50.

^h^ We used the WHO WHR ratio cutoffs; WHR ≥ 0.90 (men), WHR ≥ 0.85 (women). Only 2017–2018 data were available for hip circumference.

^i^ We used the Lancet Commission definition of at least one anthropometric criterion in addition to BMI; people were considered to have excess adiposity if they met obesity criteria per BMI in addition to WC or WHtR.

^j^ We used the Lancet Commision definition of at least two anthropometric criteria; people were considered to have excess adiposity if they had excess adiposity confirmed by WC and WHtR (≥ 0.5 per NICE). We did not include WHR data in composite metrics given limited data availability for 2017–2018 only.

LC criteria showed varying performance depending on the combination of measures. The BMI plus ≥ 1 waist‐based measure criterion had sensitivity of 74.6% and specificity of 82.2%. Meeting ≥ 2 waist‐based measures, regardless of BMI, produced 88.5% sensitivity and 66.8% specificity, with PPV and NPV similar to those of the NHLBI WC definition.

Table 3 presents diagnostic performance across race/ethnicity and sex. For CDC BMI thresholds, sensitivity ranged from 38.4% in non‐Hispanic Asian adults to 87.9% in non‐Hispanic Black adults. In comparison, specificity ranged from 73.3% in non‐Hispanic Black adults to 93.3% in non‐Hispanic Asian adults. Race‐based BMI thresholds increased sensitivity among Asian adults (62.3%) with a corresponding specificity of 83.8%, whereas values remained relatively unchanged for other racial/ethnic groups compared to CDC BMI thresholds. Measures of central adiposity showed higher sensitivity and lower specificity across groups. For NHLBI WC thresholds, sensitivity exceeded 90% in non‐Hispanic White and non‐Hispanic Black adults and was 57.6% in Asian adults, who had specificity of 86.5%. IDF WC thresholds produced sensitivity ≥ 98% in most groups and specificity ranging from 33.4% in Mexican adults to 51.4% in Asian adults. NICE WHtR thresholds produced sensitivity > 99% across most racial/ethnic groups, with specificityranging from 17.1% in Mexican adults to 39.8% in Asian adults. WHO WHR thresholds yielded sensitivity of 82.9%–94.2% and specificity of 26.6%–53.7% across groups.

**TABLE 3:** Diagnostic test performance of different obesity measures for excess adiposity confirmed by DEXA by race/ethnicity and sex.

Obesity measures^a^ ^,^ ^b^ (*N* = 10,747)	Sensitivity, %	Specificity, %	PPV, %	NPV, %
CDC BMI thresholds^c^	74.62	82.18	67.02	86.96
Race/ethnicity				
Mexican	79.69	74.89	67.64	84.84
Other Hispanic	75.50	78.91	63.57	86.86
NH White	73.76	84.43	69.71	86.89
NH Black	87.85	73.28	59.65	93.06
NH Asian	38.38	93.32	62.48	83.94
Other/multiracial	72.86	76.01	61.36	84.27
Sex				
Male	69.99	82.01	65.72	84.72
Female	79.44	82.35	68.27	89.34
Race‐based BMI thresholds^d^	75.63	81.51	66.51	87.32
Race/ethnicity				
Mexican	79.69	74.89	67.64	84.84
Other Hispanic	75.50	78.91	63.57	86.86
NH White	73.76	84.43	69.71	86.89
NH Black	87.85	73.28	59.65	93.06
NH Asian	62.32	83.83	52.77	88.47
Other/multiracial	72.86	76.01	61.36	84.27
Sex				
Male	71.22	81.19	65.11	85.13
Female	80.21	81.83	67.85	89.64
NHLBI WC thresholds^e^	88.54	66.74	56.38	92.31
Race/ethnicity				
Mexican	88.15	60.84	59.72	88.63
Other Hispanic	85.16	69.19	57.39	90.54
NH White	90.34	66.10	56.41	93.37
NH Black	93.59	62.23	52.71	95.57
NH Asian	57.59	86.53	55.34	87.56
Other/multiracial	87.57	63.64	55.74	90.73
Sex				
Male	81.49	80.26	67.05	89.79
Female	95.87	53.11	49.42	96.42
IDF race‐based WC thresholds^f^	98.47	39.93	44.31	98.17
Race/ethnicity				
Mexican	98.79	33.36	49.40	97.66
Other Hispanic	98.29	40.81	44.73	97.99
NH White	98.54	38.73	43.85	98.20
NH Black	99.80	43.13	44.11	99.79
NH Asian	92.69	51.41	35.61	96.04
Other/multiracial	99.40	43.12	47.74	99.27
Sex				
Male	97.19	53.18	50.57	97.46
Female	99.81	26.57	39.38	99.65
Swainson WHtR data (WHtR ≥ 0.53)^g^	99.52	32.96	41.88	99.30
Race/ethnicity				
Mexican	99.85	17.09	44.23	99.43
Other Hispanic	99.73	25.36	39.44	99.49
NH White	99.47	34.90	42.60	99.27
NH Black	100.00	35.94	41.25	100.00
NH Asian	97.26	39.76	31.88	98.04
Other/multiracial	100.00	34.30	44.31	100.00
Sex				
Male	99.37	33.14	42.28	99.06
Female	99.69	32.77	41.48	99.55
WHO WHR thresholds^h^	90.78	42.10	44.39	89.96
Race/ethnicity				
Mexican	94.20	26.58	49.59	85.67
Other Hispanic	90.93	39.36	42.07	89.96
NH White	92.04	43.63	43.04	92.21
NH Black	82.87	53.66	47.86	85.92
NH Asian	82.95	36.98	34.80	84.25
Other/multiracial	93.02	40.70	56.96	87.37
Sex				
Male	96.59	37.36	46.18	95.16
Female	84.40	46.45	42.34	86.47
Lancet Commission criteria^i^ ^,^ ^j^				
≥ 1 anthropometric measure + BMI^g^	74.62	82.18	67.02	86.96
Race				
Mexican	79.69	74.89	67.64	84.84
Other Hispanic	75.50	78.91	63.57	86.86
NH White	73.76	84.43	69.71	86.89
NH Black	87.85	73.28	59.65	93.06
NH Asian	38.38	93.32	62.48	83.94
Other/multiracial	72.86	76.01	61.36	84.27
Sex				
Male	69.99	82.01	65.72	84.72
Female	79.44	82.35	68.27	89.34
≥ 2 anthropometric measures^h^	88.54	66.78	56.40	92.31
Race				
Mexican	88.15	60.84	59.72	88.63
Other Hispanic	85.16	69.19	57.39	90.54
NH White	90.34	66.15	56.45	93.38
NH Black	93.59	62.23	52.71	95.57
NH Asian	57.59	86.53	55.34	87.56
Other/multiracial	87.57	63.64	55.74	90.73
Sex				
Male	81.49	80.26	67.05	89.79
Female	95.87	53.18	49.46	96.43

Abbreviations: DEXA, dual‐energy X‐ray absorptiometry; NPV, negative predictive value; PPV, positive predictive value; WC, waist circumference WHtR, waist to height ratio; WHR, waist to hip ratio.

^a^ Excess adiposity was defined as a high percentage of body fat on DEXA: ≥ 30% (men), ≥ 42% (women).

^b^ All analyses were conducted using NHANES sample weights and variance estimates.

^c^ We used the CDC definition of obesity, BMI ≥ 30.

^d^ We used race‐based modifications per WHO recommendations; BMI ≥ 27.5 (Asians), BMI ≥ 30 (others).

^e^ We used the NHLBI WC cutoffs: WC ≥ 102 cm (men), WC ≥ 88 cm (women).

^f^ We used the IDF WC cutoffs; WC ≥ 90 cm (Asian men), ≥ 94 cm (other men), ≥ 80 cm (women).

^g^ We used the NICE WHtR cutoffs; WHtR ≥ 0.50.

^h^ We used the WHO WHR cutoffs; WHR ≥ 0.90 (men), WHR ≥ 0.85 (women). Only 2017–2018 data were available for hip circumference.

^i^ We used the Lancet Commission definition of at least one anthropometric criterion in addition to BMI; people were considered to have excess adiposity if they met obesity criteria per BMI in addition to WC or WHtR.

^j^ We used the Lancet Commission definition of at least two anthropometric criteria; people were considered to have excess adiposity if they had excess adiposity confirmed by WC and WHtR (≥ 0.5 per NICE). We did not include WHR data in composite metrics given limited data availability for 2017–2018 only.

Sex‐stratified results showed higher sensitivity and lower specificity among women for nearly all definitions. For NHLBI WC thresholds, sensitivity was 95.9% in women and 81.5% in men, while specificity was 53.1% in women and 80.3% in men. Similar patterns were observed for LC definitions: the BMI plus ≥ 1 waist measure criterion yielded sensitivity ranging from 38.4% amaong Asian adults to 87.9% among Black adults, and the ≥ 2 waist‐based measures criterion produced values closely aligned with those of NHLBI WC thresholds across race/ethnicity and sex strata.

**FIGURE 2:**
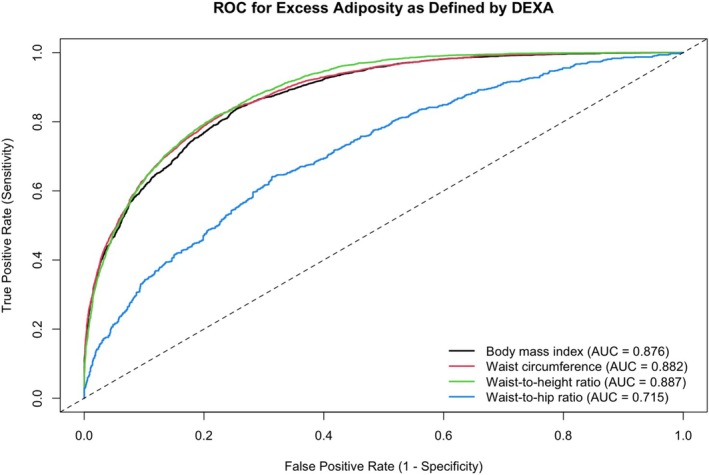
Receiver operating characteristic (ROC) curves for various anthropometric measures predicting excess adiposity defined by DEXA. [Color figure can be viewed at wileyonlinelibrary.com]

Figure 2 displays the ROC curves for BMI, WC, WHtR, and WHR in relation to DEXA‐defined excess adiposity. The area under the curve (AUC) values were 0.876 for BMI, 0.882 for WC, 0.887 for WHtR, and 0.715 for WHR. The curves for BMI, WC, and WHtR showed similar trajectories, while WHR showed a lower overall AUC.

## 4 Discussion

In this nationally representative sample of US adults aged 20–59 years, waist‐based measures of obesity demonstrated greater sensitivity but lower specificity than BMI for identifying DEXA‐defined excess adiposity. NICE WHtR, Swainson WHtR, and WHO WHR thresholds showed similar patterns with high sensitivity but low specificity. Additionally, race‐based BMI thresholds yielded improved sensitivity among Asian adults (62.3% vs. 38.4%) compared to race‐neutral BMI thresholds, with specificity of 83.8%. ROC analyses showed comparable discrimination for BMI, WC, and WHtR but lower discrimination for WHR.

LC criteria demonstrated intermediate performance depending on the combination of measures. Requiring BMI plus ≥ 1 waist‐based measure achieved 73.5% sensitivity and 84.7% specificity, similar to CDC BMI thresholds. In contrast, meeting ≥ 2 waist‐based measures yielded 88.5% sensitivity and 66.8% specificity, closely mirroring the NHLBI WC performance. Across racial/ethnic groups, waist‐based measures consistently improved sensitivity relative to BMI alone, although specificity was particularly low among Mexican and Asian adults. Sex‐stratified analyses revealed higher sensitivity and lower specificity among women compared with men for nearly all definitions of the condition.

These findings confirm prior reports that waist‐based measures are more sensitive than BMI for detecting excess body fat, particularly among populations with lower BMI or higher abdominal fat accumulation [2, 3, 14, 15, 16]. Race‐based BMI thresholds improved detection among Asian adults but had minimal effect on other groups, suggesting that universal WC or WHtR cut points may be more effective for screening [17]. Our results also demonstrate that composite criteria, such as those proposed by the LC, are similar to BMI and WC alone in identifying excess adiposity [2].

This study has limitations. First, our analysis was restricted to US adults aged 20–59 years and may not be generalizable to older populations. Second, while DEXA is a widely accepted reference standard for body composition, it may underestimate abdominal adiposity relative to computed tomography (CT), although agreement between DEXA and CT is high [3]. Third, NHANES response rates were generally under 70%. However, sampling weights and survey design variables were applied to account for nonresponse and complex sampling.

Overall, our findings support the use of waist‐based measures of obesity, either alone or in combination with BMI, to more accurately identify individuals with excess adiposity across diverse populations. Waist‐based measures may thus be more suitable for clinical and public health screening, particularly in populations where BMI alone may underestimate adiposity‐related risk.

## Author Contributions

Concept and design, acquisition, analysis, or interpretation of data, drafting of the manuscript, critical review of the manuscript for important intellectual content, and statistical analysis: all authors. F.C.S.: administrative, technical, or material support and supervision. All authors had full access to all the data in the study and take responsibility for the integrity of the data and the accuracy of the data analysis.

## Funding

This work was supported by National Institutes of Health grants NIDDK P30 DK040561, U24 DK132733, and UE5 DK137285 (F.C.S.).

## Conflicts of Interest

The authors declare no conflicts of interest. F.C.S. served on the Lancet Commission on the Definition and Diagnosis of Clinical Obesity.

## Data Availability

All data are available on websites published by the National Center for Health Statistics, Centers for Disease Control and Prevention (https://www.cdc.gov/nchs/nhanes/index.html).
